# Parathyroid hormone receptor 1 (PTHR1) is a prognostic indicator in canine osteosarcoma

**DOI:** 10.1038/s41598-020-58524-3

**Published:** 2020-01-31

**Authors:** Awf A. Al-Khan, Judith S. Nimmo, Mourad Tayebi, Stewart D. Ryan, James O. Simcock, Raboola Tarzi, Charles A. Kuntz, Eman S. Saad, Michael J. Day, Samantha J. Richardson, Janine A. Danks

**Affiliations:** 1 RMIT University, School of Health and Biomedical Sciences, Bundoora, 3083 Australia; 2 Australian Specialised Animal Pathology Laboratory, Mulgrave, 3170 Australia; 3 Western Sydney University, School of Medicine, Campbelltown, 2560 Australia; 4 The University of Melbourne, Faculty of Veterinary & Agricultural Sciences, Translational Research and Animal Clinical Trial Study Group (TRACTS), Werribee, 3030 Australia; 5 Southpaws Veterinary Hospital, Department of Surgery, Moorabbin, 3189 Australia; 6 Murdoch University, School of Veterinary and Life Sciences, Murdoch, 6150 Australia; 7 RMIT University, School of Science, Bundoora, 3083 Australia; 8 The University of Melbourne, Department of Medicine, Austin Health, Heidelberg, 3084 Australia; 9 Present Address: Suhar Hospital, Department of Pathology, Suhar, 311 Oman

**Keywords:** Parathyroid diseases, Surgical oncology

## Abstract

Osteosarcoma (OS) is the most common malignant primary bone tumour in humans and dogs. Several studies have established the vital role of parathyroid hormone-related protein (PTHrP) and its receptor (PTHR1) in bone formation and remodeling. In addition, these molecules play a role in the progression and metastasis of many human tumour types. This study investigated the expression of PTHR1 and PTHrP in canine OS tissues and assessed their prognostic value. Formalin-fixed, paraffin-embedded tissue samples from 50 dogs diagnosed with primary OS were immunolabeled with antibodies specific for PTHR1 and PTHrP. The immunostaining intensity of tumours from patients with OS was correlated with survival time. Both PTHR1 and PTHrP were detected in all OS samples (n = 50). Dogs with OS tumours showing high immunostaining intensity for PTHR1 (n = 36) had significantly shorter survival times (p = 0.028, Log Rank; p = 0.04, Cox regression) when compared with OS that had low immunostaining intensity for PTHR1 (n = 14).PTHrP immunostaining intensity did not correlate with survival time (p > 0.05). The results of this study indicate that increased expression of PTHR1 antigen in canine OS is associated with poor prognosis. This suggests that PTHR1 may be useful as a prognostic indicator in canine OS.

## Introduction

Osteosarcoma (OS) is a malignancy that originates from bone-forming mesenchymal cells^1^. It is the most prevalent type of primary bone cancer in both humans and dogs^2–5^. Canine OS represents 85–98% of all canine primary bone cancers and occurs more commonly in appendicular skeleton (75%)^6^. In the USA, more than 10,000 cases of canine OS are reported every year^7^.

In the last 35 years, there has been little improvement in the treatment of human OS or its prognosis after treatment with surgery and chemotherapy, especially for patients with metastatic OS^8^. It has been found that the median survival times of dogs suffering from OS and receiving standard care (surgery and adjuvant chemotherapy) is from three months to one year and that less than 20% will be alive more than two years after diagnosis^9^. So, there is a need to identify indicators for early diagnosis and prediction of prognosis in OS which may assist in improvement of patient survival.

Parathyroid hormone-related protein (PTHrP) was discovered as the factor responsible for causing hypercalcemia of malignancy in some tumours^10^. Parathyroid hormone (PTH) and PTHrP share a similar amino acid sequence in the N-terminal region^10^. That allows the two to activate a common G-protein coupled receptor known as PTH/PTHrP receptor (PTHR1)^11^. PTHrP and its receptor are highly conserved amongst all vertebrates^12^.

PTHrP is produced in many normal tissues where it acts as an autocrine/paracrine regulator of cell growth, development and differentiation^13^. In addition, PTHrP has been localised in numerous human cancers including breast cancers^14^, neuroendocrine cancers^15^, prostate cancers^16^, squamous cell carcinoma of skin^17^, pancreatic adenocarcinoma^18^, intrahepatic cholangiocarcinoma^19^ and OS^20^. Several studies showed that PTHrP is also produced by canine tumours such as thyroid carcinoma, multiple myeloma, anal gland adenocarcinomas^21^, mammary gland carcinoma^22^, thyroid carcinoma^23^, histiocytic sarcoma and renal cell carcinoma^24^.

Recently, Hastings *et al*.^25^ revealed that increased expression of PTHrP was associated with poor prognosis in lung carcinoma. In addition, secretion of PTHrP by tumour cells may be involved in metastasis and in the regulation of primary breast tumour growth^26^.

PTHR1 is a member of the class B G-protein coupled receptor (GPCR) family^27^. Several studies carried out using human tissues^28^, mice^29^ and cell lines^30^ have noted the association between PTHrP/PTHR1 expression and OS. Yang and his group^30^ suggested that over-expression of *PTHR1* may stimulate human OS progression via formation of a more aggressive phenotype. Furthermore, Ho and colleagues^29^ demonstrated that knockdown of *PTHR1* in murine OS cells reduced growth and invasion and increased tumour differentiation. Recently, a study has shown that PTHrP is an essential factor for initiation, hyperproliferation and maintenance of murine p53-deficient OS^31^. The authors found that PTHrP activated PTHR1 and increased cAMP leading to cAMP response element-binding protein 1 (CREB1) phosphorylation and transcriptional activation^31^. Walia *et al*.^31^ concluded that PTHrP-cAMP-CREB1 axis was crucial for initiation of murine OS in p53-deficient osteoblasts.

Previously, it was shown that if PTHR1 expression *in vivo* was decreased, then OS had enhanced mineralisation and differentiation^29^. In addition, Ho *et al*.^29^ found that levels of PTHrP were higher in the osteoblastic histological subtype of murine OS compared to the less aggressive subtype. However, PTHR1 and PTHrP were not investigated in canine OS and there is no data about their association with survival time.

To further evaluate the prognostic value of PTHR1 and PTHrP in OS, their expression was investigated in the naturally occurring canine OS. The study also examined whether such expression was associated with survival.

## Results

### Clinical and epidemiological data

Fifty canine primary OS samples (ASAP-50) were collected prospectively from the Australian Specialised Animal Pathology (ASAP) laboratory and the data were obtained independently (Table 1). The age ranged from 1 to 15 years with a mean and median age of 9 years and males (30 dogs, 60%) were predominant. Thirty dogs (60%) were of a pure breed and 20 (40%) were of mixed breed. There were 42 (84%) dogs of large or medium breeds and eight (6%) of small breeds. The frequency of the top two represented breeds was Labrador (five dogs, 10%) and Rottweiler (five dogs, 10%). Tumours were located mostly in the humerus (13 dogs, 26%), jaw (eight dogs, 16%) and radius (seven dogs, 14%) (Table 1).

**Table 1:** Clinicopathological data and immunostaining intensity of PTHR1 and PTHrP for those cases collected from ASAP laboratory with survival time (ASAP-50).

#	Breed	Sex	Age	Body part	Tumour grade	Survival time (days)	PTHR1 immuno-staining intensity	PTHrP immune-staining intensity
1	Staffordshire Bull Terrier	F	12	Humerus	1	28*	Low	Low
2	Greyhound	M	10	Tibia	1	90	High	High
3	Rottweiler	M	1	Humerus	1	114	High	Low
4	Boxer Cross	M	11	Femur	1	458	Low	Low
5	Mastiff	F	7	Rib	1	14*	Low	Low
6	Bulldog	M	10	Tibia	1	193	Low	Low
7	Labrador Retriever	M	8	Radius	1	702	Low	Low
8	Giant Schnauzer	M	7	Radius	2	380	High	High
9	Boxer	F	10	Rib	2	240*	Low	Low
10	German Shepherd Dog	F	11	Radius	2	485	High	High
11	Rottweiler Cross	F	8	Jaw	1	83*	High	High
12	Spoodle	M	10	Rib	2	71*	High	Low
13	Schnauzer Cross	M	11	Rib	2	4*	Low	Low
14	Doberman	F	10	Tibia	1	240	High	Low
15	Rottweiler	M	8	Humerus	1	150*	High	Low
16	Rottweiler	F	9	Jaw	1	157*	High	Low
17	Golden Retriever	M	8	Humerus	1	65*	Low	Low
18	Golden Retriever	M	14	Femur	2	115*	High	High
19	Rottweiler Cross	M	9	Ilium	1	1*	High	Low
20	Doberman	M	9	Radius	1	104*	Low	Low
21	Labrador	F	7	Radius	2	277	Low	Low
22	Jack Russell Terrier	M	8	Jaw	2	21*	High	Low
23	Rottweiler	F	4	Humerus	2	96	High	Low
24	Blue Heeler	M	12	Tibia	1	150*	High	High
25	Golden Retriever	M	13	Jaw	1	180*	Low	High
26	Mastiff Cross	M	6	Femur	2	470	Low	Low
27	Cairn Terrier	F	12	Humerus	1	155	High	High
28	Boxer Cross	F	15	Jaw	1	43*	High	High
29	Jack Russell Terrier	F	9	Scapula	2	27*	High	High
30	Labrador	M	11	Humerus	1	578	High	High
31	Cavalier Cross	M	10	Humerus	2	26	High	High
32	Labrador	M	13	Jaw	2	21*	High	High
33	Border Collie	M	6	Femur	2	17*	High	Low
34	Labradoodle	M	3	Rib	2	11*	High	High
35	Pointer Cross	F	9	Radius	1	17*	High	Low
36	Rottweiler Cross	F	9	Humerus	2	270	High	Low
37	Border Collie Cross	F	13	Scapula	2	135*	Low	Low
38	American Staffordshire Bull Terrier	M	12	Vertebrae	1	54*	High	Low
39	White Swiss Shepherd Dog	F	9	Ilium	2	3*	High	High
40	Maltese Cross	M	13	Jaw	2	47*	High	High
41	SBT Cross	F	9	Humerus	2	76	High	High
42	Alaskan Malamute	M	6	Radius	1	82	High	Low
43	Rottweiler	F	11	Tibia	2	288	High	High
44	Curly Coated Retriever	F	11	Humerus	1	90	High	High
45	Boxer	F	7	Skull	1	24*	High	High
46	Labrador	M	8	Humerus	2	14*	High	High
47	Jack Russell Terrier	M	14	Jaw	2	154*	High	High
48	Labrador	M	7	Femur	1	7*	High	High
49	Greyhound	M	11	Femur	1	655	Low	High
50	Labradoodle	M	7	Humerus	1	142*	High	High

*Cases excluded from the larger ASAP-50 group of dogs with OS to make a homogenous group (ASAP-20). Excluded dogs had no surgical or no chemotherapy, or had pulmonary metastasis at presentation, or presented with OS localised in axial parts, or still alive at the date of data collection, or died because of post-surgical complications; SBT, Staffordshire Bull Terrier; F, female; M, male.

### Histopathology

The histopathological review classified osteoblastic OS (33 dogs, 66%) as the most common subtype of canine OS while, chondroblastic (11 dogs, 22%) and fibroblastic (six dogs, 12%) OS were less frequent (Supplementary Table S1). Twenty-seven canine OS tumours (54%) were graded as G1 and 23 tumours (46%) as G2 (Table 1).

### PTHR1 immunostaining intensity in canine OS

**Figure 1:**
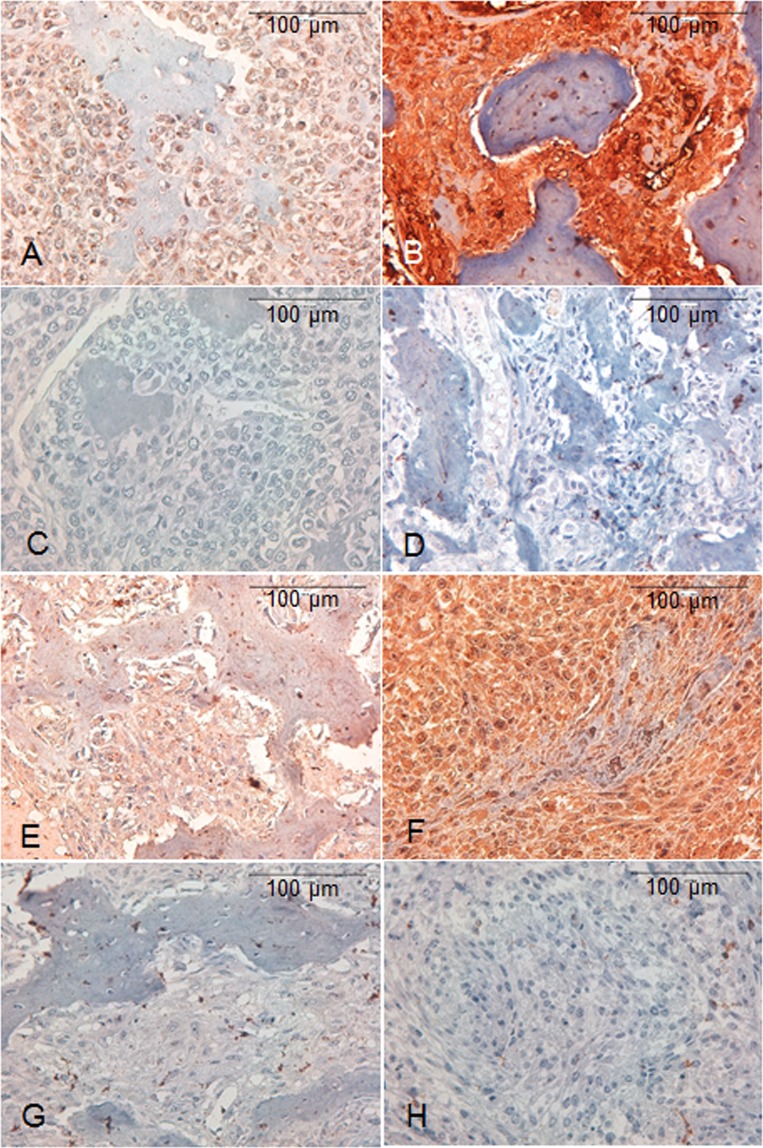
Immunohistochemical staining for PTHR1 and PTHrP in canine OS. Positive and negative cells could be seen in both the tumour and the osteoid areas. (**A**) Neoplastic cells are characterised by low cytoplasmic plus nuclear immunostaining intensity for PTHR1. (**B**) Neoplastic cells are characterised by high cytoplasmic plus nuclear immunostaining intensity for PTHR1. (**C, D**) Non-immune control for the above cases. Neoplastic cells are characterised by absent cytoplasmic or nuclear immunolabelling for PTHR1. (**E**) Neoplastic cells display low cytoplasmic immunostaining intensity for PTHrP. (**F**) Neoplastic cells display high cytoplasmic plus nuclear immunostaining intensity for PTHrP. (**G**, **H**) Non-immune control for the above cases. Neoplastic cells are characterised by absent cytoplasmic or nuclear immunolabelling for PTHrP. Immunohistochemistry. Mayer’s haematoxylin counterstaining.

PTHR1 was detected in all cases (n = 50). PTHR1 showed low immunostaining intensity in 14 (28%) cases (Fig. 1A, C: non-immune control) and high immunostaining intensity in 36 (72%) cases (Fig. 1B, D: non-immune control) (Table 1). Cytoplasmic plus nuclear localisation of PTHR1 was observed in the neoplastic cells in all cases. There was no significant association between PTHR1 immunostaining intensity and the clinicopathological data (age, gender, breed, tumour grade) (p > 0.05, Chi-Square test).

### PTHrP immunostaining intensity in canine OS

PTHrP was localised in the all canine OS sections (n = 50). Low immunostaining intensity of PTHrP was detected in 25 (50%) cases (Fig. 1E) and high immunostaining intensity in 25 (50%) cases of canine OS (Fig. 1F) (Table 1). PTHrP was localised to the cytoplasm of neoplastic cells in 33 (66%) cases and to the nucleus plus the cytoplasm in 17 (34%) cases. There was no significant difference in PTHrP immunostaining intensity between dogs with different ages at diagnosis, genders, breeds and tumour grades (*P* > 0.05, Chi-Square test). PTHR1 and PTHrP immunolabelling of normal canine kidney tissue revealed strong cytoplasmic positivity in proximal and distal tubules, in contrast to weak immunolabelling in glomeruli^28,32^.

Supplementary Figs. S1 and S2 show western blot analysis of PTHrP expression in canine OS.

### High immunostaining intensity of PTHR1 in canine OS (ASAP-50) correlates with decreased survival time and may act as a prognostic indicator

**Figure 2:**
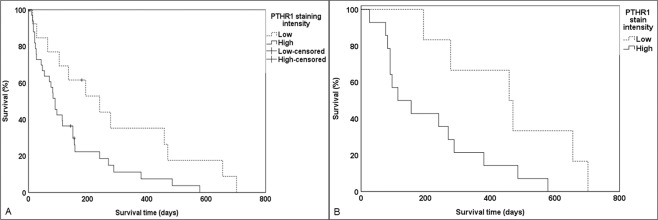
Kaplan-Meier survival analysis of canine OS with high and low immunostaining intensity of PTHR1. (**A**) Dogs (ASAP-50 group) with tumours showing high immunostaining intensity of PTHR1 (139 ± 27 days, *n* = 36) had shorter survival times compared to dogs with tumours showing low immunostaining intensity (290 ± 68 days, *n* = 14) (*P* = 0.028, Log Rank test; *n* = 50). (**B**) Dogs (ASAP-20 group) with tumours showing PTHR1 high immunostaining intensity (212 ± 45 days, *n* = 14) had shorter survival times compared to dogs with tumours showing low immunostaining intensity (459 ± 82 days, *n* = 6) (*P* = 0.030, Log Rank test; *n* = 20).

To investigate the relationship between immunostaining intensity of PTHR1 and the survival times of dogs with OS, the survival times for dogs with OS showing high immunostaining intensity of PTHR1 were compared to survival times for those showing low immunostaining intensity. Dogs with OS showing high immunostaining intensity for PTHR1 had shorter average survival times (mean = 139 ± 27 days) compared to those with low immunostaining intensity (mean = 290 ± 68 days). This difference was significant (p = 0.028, Log Rank; n = 50; 95% confidence interval (CI), 127.2–240.4) (Fig. 2A).

There was no significant difference between PTHrP immunostaining intensity and survival times (p = 0.782, Log Rank; n = 50, 95% CI, 127.2–240.4).

The univariate Cox regression analysis identified that high immunostaining intensity of PTHR1 in dogs with OS (p = 0.032, n = 50; univariate Cox regression, 95% CI, 0.218–0.935) was predictor for poor prognosis. Other variables such as expression of PTHrP, age, gender, breed and tumour grade did not show any significant prognostic value (p > 0.05). In addition, multivariate Cox regression analysis showed that immunostaining intensity of PTHR1 is an independent significant prognostic indicator for canine OS (p = 0.040, n = 50; multivariate Cox regression, 95% CI, 0.172–0.961).

### High PTHR1 immunostaining intensity correlates with reduced survival time and acts as a prognostic indicator for the smaller group of dogs with OS (ASAP-20)

In order to minimise variation in the population and validate the findings presented above, dogs that had no surgical (24 dogs) or no chemotherapy treatments (26 dogs), or had pulmonary metastasis at presentation (four dogs), or presented with OS localised in axial parts (15 dogs), or were still alive at the date of data collection (three dogs), or died because of post-surgical complications (four dogs) were excluded from survival analyses (Table 1). The remaining 20 dogs (40%) with appendicular OS (ASAP-20) had limb amputation and then went through adjuvant chemotherapy using cisplatin or carboplatin as a single or multiple dose, had no pulmonary metastasis and died due to OS. The survival times for dogs with OS showing high PTHR1 immunostaining intensity and for those with OS showing low PTHR1 immunostaining intensity were compared.

The Log Rank test showed that there was a significant difference in the survival time between groups of dogs with appendicular OS showing high and low immunostaining intensity of PTHR1 (p = 0.030; n = 20; 95% CI: 194.6–377.9) (Fig. 2B). Dogs with appendicular OS showing high PTHR1 immunostaining intensity had shorter average survival times (mean = 212 ± 45 days) compared to those with low immunostaining intensity (mean = 459 ± 82 days) (Fig. 2B).

Moreover, the prognostic value of PTHR1 immunostaining intensity for overall survival in the smaller group of dogs with appendicular OS (ASAP-20) was analysed using the univariate Cox regression analysis on different clinicopathological parameters, including breed size, sex, age, tumour grade and PTHrP immunostaining intensity. The analysis showed that PTHR1 immunostaining intensity was the only parameter that influenced survival rates (p = 0.040, univariate Cox regression; n = 20; 95% CI: 0.099–0.948). Other variables including breed size, sex, age, tumour grade and PTHrP immunostaining intensity did not show any significant prognostic value (p > 0.05).

A multivariate Cox regression analysis was used to further evaluate the prognostic value of PTHR1 immunostaining intensity in canine OS prognosis. The analysis revealed that PTHR1 immunostaining intensity was an independent prognostic factor for overall survival (p = 0.040, multivariate Cox regression; n = 20; 95% CI: 0.172–0.961). Thus, the findings also suggest that the immunostaining intensity of PTHR1 is significantly linked with the prognosis of canine OS.

## Discussion

OS is the most prevalent primary bone cancer in humans and dogs^2–5^. Despite the current treatment consisting in surgery and adjuvant chemotherapy, OS bears a poor prognosis^33^. Identification and characterisation of novel markers that could assess possible survival time might allow for better categorisation of patients for risk-based treatment. PTHR1 and PTHrP have not been investigated as prognostic indicators in either canine or human OS and until now the presence of PTHR1 and PTHrP has not been investigated in naturally occurring canine OS.

The aim of this study was to determine the expression of PTHR1 and PTHrP in canine OS tissues at the cellular level and to investigate whether this immunostaining intensity had any prognostic value. PTHR1 was detected in 100% of canine OS. To better evaluate the prognostic value of PTHR1and PTHrP, the survival times of dogs with high immunostaining intensity tumours were compared with those that had low immunostaining intensity tumours. Univariate and multivariate analyses revealed that immunolabelling of PTHR1 is a significant prognostic indicator in dogs with OS. Dogs with high immunostaining intensity OS tumours for PTHR1 were characterised by a significantly shorter survival time than those with low immunostaining intensity tumours. In addition, no significant correlation was observed between immunostaining intensity of PTHrP and survival times for dogs with OS. To the best of our knowledge, this is the first study to show the negative correlation of PTHR1 in canine OS with survival time.

PTHR1 was first localised at the cellular level in human OS and 17 other tumours and their tissues of origin using Western blot and IHC in 2010^28^. Lupp and others^28^ found that PTHR1 was present in 50% (*n* = 4) of human OS. In addition, a recent study showed that *PTHR1* was expressed in mouse OS cells^29^. In the same study, Ho and colleagues^29^ found that knockdown of *PTHR1* in murine OS cells increased tumour differentiation and reduced cell growth and invasion. Furthermore, a previous research revealed that overexpression of *PTHR1* in human OS xenografts was correlated with increased proliferation, cell migration and invasion^30^. However, expression of *PTHR1* was also detected in human OS cell lines (OS160, OS164, OS166, OS187 and OS191) derived from patients, but at a low level^30^. Moreover, these authors found that tissue of metastatic human OS had high expression of *PTHR1* mRNA compared with the tissue from the primary tumour^30^. Collectively, these studies support our hypothesis that increased amounts of PTHR1 in OS may be correlated with poor prognosis.

Our findings also demonstrated that similar to PTHR1, PTHrP was detected in 100% of canine OS. The current study found that dogs with low immunostaining intensity tumours for PTHrP did not have a significantly longer survival time when compared with dogs that had high immunostaining intensity tumours.

Number of studies in rat and mouse OS cell lines reported the expression of *PTHrP*^29,34^. Yang and colleagues^30^ found that *PTHrP* mRNA was not detected in aggressive human OS xenografts. Although we did not examine gene expression, one could extrapolate that the increase in protein might be a result of increased gene expression. It has been shown that overexpression of the *PTHrP* gene is correlated with reduced cell proliferation using a murine OS cell line^35^. This was supported by work that overexpression of *PTHrP* in a rat OS cell line was also associated with decreased growth rate^36^.

On the other hand, a study was conducted using the Saos-2 human OS cell line^37^, in which *PTHrP*-induced tumour cells became resistant to chemotherapy via the inhibition of major apoptosis signaling pathways by blocking the death receptor and mitochondria-mediated apoptosis signaling. In addition, Berdiaki and colleagues^38^ found that PTHrP stimulates migration of MG-63 and Saos 2 human OS cell lines. The last two studies^37,38^ suggested that increased amounts of PTHrP may be associated with shorter survival time. Recently, Walia and others^31^ revealed that PTHrP is crucial to initiate OS in murine p53-deficient osteoblasts. The researchers showed that PTHrP binds to PTHR1 which stimulates production of cAMP, resulting in phosphorylation of CREB1 and activation of transcription in p53-deficient OS^31^. This may explain the detection of high immunostaining intensity of PTHrP and PTHR1 in the most of OS tissues in the present study.

The present study demonstrated cytoplasmic and nuclear localisation of both PTHR1 and PTHrP. It has been shown that PTHR1 is transported to the nucleus after binding to the transport regulatory proteins, importin α1 and importin β^39^, while PTHrP binds to importin β^40^. Pickard *et al*.^39^ found that PTHR1 was overexpressed in the nucleus during early interphase stage (G0/G1, S, and G2 phases) of the cell cycle using MC3T3-E1 mouse non-transformed osteoblasts, SaOS-2 human OS and ROS 17/2.8 rat OS cell lines. In these early stages, the DNA is known to be open to transcriptional activity comparing to the late stages where the DNA is compact and transcriptional activities are very less and the immunostaining intensity of PTHR1 is low^39^. In tumours, the abundant nuclear localisation of PTHR1 could be explained by the increased rate of neoplastic cell mitosis and most of these cells are at the early stages.

Recently, it was suggested that PTHrP stimulates matrix mineralisation and proliferation of osteoblasts through three mechanisms: an autocrine/paracrine signal-peptide/PTHR1-dependent mechanism, an intracrine nuclear localisation signal-dependent mechanism and mixed mechanism^41^. Even though associated with stimulating proliferation and reducing apoptosis in certain cell types, the exact role of PTHrP in the nucleus/nucleolus still unclear^42^.

Furthermore, Walkley *et al*.^43^ suggested the pathway of PTHR1 in OS carcinogenesis. Normally, the PTHR1 located on the surface of normal osteoblasts is activated via binding to PTHrP. Activation of PTHR1 resulted in the synthesis of cyclic adenosine 3′,5′-monophosphate (cAMP) from ATP by adenylyl cyclase. Thus, cAMP induces the release of cAMP-dependent protein kinase A (PKA) from its α regulatory subunit of PKA type 1 (PRKAR1A). After that, triggered PKA enter into the nucleus to phosphorylate and activate cAMP response element-binding protein 1 (CREB1). This leads to activate the signaling of target gene downstream of PTHR1^43^. In OS, numerous defects in the PTHrP-PTHR1-PKA pathway rise the activity of PKA pathway including increased expression of *Prkaca* gene that encodes catalytic component of PKA and amplified number of PTHR1 on the cell surface. In addition, other defects are mutations in *Prkar1a* gene and increased production of PTHrP. The mutation of *Prkar1a* gene leads to increase the activity of PKA, while increased production of PTHrP which can bind to PTHR1 and stimulate the formation of cAMP^43^. Recently, Li *et al*.^44^ proposed that the effects of PTHR1 could be mediated by triggering angiogenesis, inflammation and Wnt pathways through altering the expressions of the crucial enriched genes (*Dkk1*, *Lef1*, *Agt-CCR3*, and *Agt-CCL9*) using mouse OS cells.

The current study did not detect any significant prognostic value for other variables such as age, gender, breed or tumour grade. Similar findings were reported by some previous studies^45–49^.

The effects of the decalcification were not controlled in this study. Canine kidney (without decalcification) was used as the normal control tissue and was routinely immunostained with the canine OS samples. In our experience, decalcification lowers the amount of immunostaining seen in the tissues, but we have taken this into account during the optimization of IHC. According to the available data in the current study, the duration of decalcification had limited effect on the staining intensity of PTHR1 and PTHrP.

In the current study, the cause of death was assumed to be OS unless it was obviously otherwise. Cause of death determination is complex. The standard for most of cancer studies is to assume the worst-case scenario. This is reasonable as OS has a high metastatic rate. In most cases, metastases were observed on repeated imaging. In a few cases, like the one assumed to have developed a brain lesion, we assumed that it was metastatic OS since this was the most reasonable assumption.

In conclusion, this study localised PTHR1 and PTHrP in canine OS. Moreover, it has demonstrated that low immunostaining intensity of PTHR1 in canine OS related to a better prognosis, while high immunostaining intensity of PTHR1 is correlated with decreased survival time and it is a significant indicator for poor prognosis.

## Methods

### Canine tumour specimens

A total of 50 formalin-fixed, paraffin-embedded (FFPE) canine OS tissues reported between 2014 and 2016 at the Australian Specialised Animal Pathology Laboratory (ASAP), Mulgrave, Victoria, Australia were available for this study (ASAP-50 group) (Table 1). These samples were collected prospectively. To minimise variation in the samples, patients with axial affected parts (24%), or had no surgical or no chemotherapy treatments (22%), or had pulmonary metastasis at presentation (6%), or still alive at the date of data collection (4%), or died because of surgery post-complication (4%) were excluded from this study. The survival times were analysed for the ASAP-50 group and for the remaining 20 patients (40%) (ASAP-20group). The samples were submitted from the following veterinary clinics/hospitals Southpaws Specialty Surgery for Animals (16 cases), Advanced Veterinary Care (three cases), Gasing Veterinary Clinic (three cases), Preston Veterinary Clinic (three cases), Glenhuntly Road Veterinary Clinic (two cases), Hampton Veterinary Hospital (two cases), Knox Veterinary Clinic (two cases), Northcote Plaza Veterinary Clinic (two cases), Prahran Veterinary Hospital (two cases), Boronia Veterinary Clinic (one case), Burvale Heights Veterinary Hospital (one case), Canterbury Veterinary Clinic (one case), Care Collingwood (one case), Carnegie Veterinary Clinic (one case),Endeavour Hills Veterinary (one case), Ferntree Gully Veterinary (one case), Maroondah Veterinary Clinic (one case), Mitcham Pet Hospital (one case), Old Sale Road Veterinary (one case), Tarwin Veterinary Group (one case), Warby Street Veterinary Hospital (one case), West Gippsland Veterinary Care (one case), Westernport Veterinary Group (one case) and Yarrambat Veterinary Hospital (one case). The diagnosis of OS was confirmed according to the World Health Organization (WHO) classification^50^ and the tumour grading was performed according to Enneking *et al*.^51^.

An application was submitted to RMIT University Animal Ethics Committee. Because the tissue collected in the normal course of treatment and not specifically for the purposes of research, it did not meet the definition of scientific procedure within the legislation of Australian code for the care and use of animals for scientific purposes, 8th edition (2013). As a result, Animal Ethics Committee approval for this project was not required and informed consent was needed from the treating veterinarians.

The 20 dogs with appendicular OS (ASAP-20) included were treated surgically (amputation) followed by adjuvant chemotherapy with a single or multiple dose of cisplatin or carboplatin (Supplementary Table S1). All canine OS specimens (ASAP-50) included were decalcified using hard decal fluid (formic acid 9.8%, hydrochloric acid 8.46%) (Australian Biostain, Traralgon, Australia) or decal fluid (formic formal saline - formaldehyde BP 4%, formic acid 33%, sodium chloride 0.85%) (Australian Biostain, Traralgon, Australia). The decalcification time depends on the hardness and size of the bone biopsy and ranges from one hour to three weeks.

Data for metastasis were gathered from veterinary clinics and dog’s histopathology diagnostic report (Supplementary Table S1). Survival data and treatment regimens were obtained by contacting the treatment veterinary clinics. Dogs were followed up through intermittent visits to the treating veterinarians but not by X-rays or blood tests.

### Immunohistochemistry

Immunohistochemical staining (IHC) was performed with PTHR1 and PTHrP antibodies on all cases (*n* = 50). The IHC method was modified from Rosol *et al*.^52^. Paraffin tissue decalcified blocks were received from the ASAP Laboratory and sections (3 μm) were cut using a microtome (RM2235, Leica, Mt Waverly, Australia). Tissue sections were fixed on positively charged glass microscope slides (Superfrost Plus) (Trajan Scientific, Ringwood, Australia). Sections were incubated in high pH retrieval solution (pH 9.0, code: 9511, CINtec, Newcastle, Australia) and placed into PT Link (Dako, Sydney, Australia) at 97 °C for 20 minutes. After that, sections were treated with 1% Triton X-100 (code: 17–1315–01, Amersham Biosciences, Uppsala, Sweden) for 30 minutes to improve antibody penetration, then 3% hydrogen peroxide (Biotech Pharmaceuticals, Laverton North, Australia) for 10 minutes to block endogenous peroxidase and 0.4% casein block (Instant Skim Milk Powder, Coles Supermarkets, Hawthorn East, Australia) for 30 minutes to block the non-specific binding sites^53^. Next, the sections were incubated with primary polyclonal antibodies (PTHR1, Sc-20749, dilution: 1 in 50, Santa Cruz, Scoresby, Australia) (PTHrP, Sc-9680, dilution: 1 in 50, Santa Cruz, Scoresby, Australia) (Envision FLEX (Antibody diluent) code: K8006, Dako, Sydney, Australia) for 1 hour and their respective secondary antibodies (rabbit anti-goat polyclonal antibody, P0449, dilution: 1 in 100, Dako, Sydney, Australia) (Dual Link System-HRP, K4061, dilution: ready to use, Dako, Sydney, Australia) for 30 minutes. Then, the sections were incubated with 3, 3′-diaminobenzidine (DAB) (Dako, Sydney, Australia) for 10 minutes to visualise and localise the targeted proteins. Finally, the slides were counterstained with Mayer’s haematoxylin (Amber Scientific, Perth, Australia), dehydrated, cleared and mounted under DPX (Grale Scientific, Ringwood, Australia). Test slides were run in duplicate with a non-immune control. Normal canine kidney tissue was used as a positive control^28,32^. Primary antibody control (no antibody) (negative reagent control, 9511, CINtec, Newcastle, Australia) was carried out on canine OS samples and canine normal kidney tissue. Immuno-stained tissue sections were microscopic assessed manually and sections with non-specific stain or with a background of moderate (+2) or strong (+3) immunostaining intensity were run again.

### Quantitative analysis of immunohistochemical staining

Digital whole slide images were produced at an absolute magnification of ×400 using the Aperio ScanScope XT Digital Pathology Slide Scanner System at Austin Health, Heidelberg, Victoria, Australia (Leica Microsystems, Sydney, Australia). Each whole slide image was annotated to select tumour areas. Localisation and intensity of immunostaining of PTHR1 and PTHrP were analysed quantitatively in six fields of representative tumour areas. The Cytoplasmic v2 spectrum analysis algorithm package was applied to calculate the percentage of immunolabeled cells and to measure the immunostaining intensity for each tumour using ImageScope analysis software.

The quality of the digital images that were produced for whole tissue sections was evaluated. The threshold for each stain was specified by the ImageScope software and the percentage area of positive staining was calculated by an algorithm based on a de-convolution method to separate the stains. Each pixel was classified according to the number of stained cells present and according to location of the positive stain (nuclear, membrane or cytoplasmic). During the selection of the six representative tumour fields for each slide, areas with osteoid or other non-tumour tissues were avoided in “marking up” the stained section. In addition, osteoid was excluded from analysis using negative pen tool.

The H-score was calculated using ImageScope viewer (version 10, Aperio Technologies, Leica Microsystems, Sydney, Australia). The H-score is calculated using the following equation: (3 × percentage of strongly immunostaining nuclei/cytoplasm + 2 × percentage of moderately immunostaining nuclei/cytoplasm + percentage of weakly immunostaining nuclei/cytoplasm). The range of the final score is from 0 to 300, where 300 equivalent to 100% of tumour cells with strong intensity^54^. Tumours with H-score from 0 to 2 were considered negative. Tumours with H-score from 2 to 150 were considered low immunostaining intensity and from 151 to 300 were considered high immunostaining intensity.

Before the Aperio scanning and the calculation of H-score, slides were manually scanned and scored. The data of manual scoring is not included in the manuscript. A modified immunoreactive score (IRS) system was used in the manual scoring method to score the IHC stained slides with anti-PTHR1 and anti-PTHrP. The IRS (0–12) of each slide was calculated by multiplying the estimated staining intensity (0, 1, 2, 3) by the estimated proportion of tumour cells with positive staining (0, 1, 2, 3, 4). Tumour immunostaining with scores from 1 to 6 were considered weakly positive and scores from 7 to 12 were considered strongly positive, while tumour with score 0 were negative. The categorization of tumours as weak and strong immunostaining intensity was similar with both manual and quantitative scoring.

### Statistical analysis

IHC results, clinicopathological data such as age, gender, breed and tumour grade, and survival time were compared and analysed using Chi-Square test (IBM SPSS Statistics 24). The Kaplan-Meier method was used to generate the survival curves. Log Rank test was performed to measure the differences in survival that occurred over the time for dogs with OS. The survival analysis was performed considering the following variables: presence of PTHR1 and PTHrP, age, gender, breed and tumour grade. In addition, univariate and multivariate analyses were performed using Cox regression (proportional hazard analysis) to analyse potential prognostic indicators. The examined indicators were expression of PTHR1 and PTHrP, age, gender, breed and tumour grade. Survival time was defined as the number of days between surgery and death because of OS. Censored data were considered those cases that died during the surgery or as a result of postoperative complications or other non-cancer related causes. The data were described using mean ± standard error, (95% confidence interval (CI)) and *P* < 0.05 was considered significant.

## Supplementary information

Supplementary information. 
