# Supplementary material for: Parathyroid hormone receptor 1 (PTHR1) is a prognostic indicator in canine osteosarcoma
Source: Sci Rep. 2020 Jan 31;10:1564. doi: 10.1038/s41598-020-58524-3 (PMC6994589; doi:10.1038/s41598-020-58524-3)
Supplement: Supplementary file 1 — Supplementary information. [file 41598_2020_58524_MOESM1_ESM.pdf]

Parathyroid hormone receptor 1 (PTHR1) is a prognostic indicator in canine osteosarcoma

Awf A. Al-Khan, Judith S. Nimmo, Mourad Tayebi, Stewart D. Ryan, James O. Simcock, Raboola Tarzi, Charles A. Kuntz, Eman S. Saad, Michael J. Day, Samantha J. Richardson, Janine A. Danks

#	Breed size	Sex	Age	Affected skeleton	Body part	Subtype of OS	Metastasis	Tumour grade	Surgical treatment	Chemotherapy	Number of doses	Survival time	Cause of death cause	PTHR1 H-score	PTHR1 staining intensity	PTHrP H-score	PTHrP staining intensity	ALP (U/L)
1	Large	Female	12	Appendicular	Humerus	Osteoblastic	No	1	No	No	0	28	Euthanized because of OS	102	Low	89	Low	N/A
2	Large	Male	10	Appendicular	Tibia	Osteoblastic	No	1	Yes	Yes	5	90	Euthanized because of OS	192	High	180	High	122
3	Large	Male	1	Appendicular	Humerus	Osteoblastic	No	1	Yes	Yes	5	114	Euthanized because of OS	183	High	107	Low	N/A
4	Large	Male	11	Appendicular	Femur	Osteoblastic	No	1	Yes	Yes	6	458	OS	71	Low	86	Low	N/A
5	Large	Female	7	Axial	Rib	Chondroblastic	No	1	No	No	0	14	OS	118	Low	102	Low	N/A
6	Large	Male	10	Appendicular	Tibia	Osteoblastic	No	1	Yes	Yes	1	193	Euthanized because of OS	127	Low	93	Low	132
7	Large	Male	8	Appendicular	Radius	Osteoblastic	No	1	Yes	Yes	4	702	OS	57	Low	78	Low	N/A
8	Large	Male	7	Appendicular	Radius	Osteoblastic	No	2	Yes	Yes	5	380	Euthanized because of OS	177	High	229	High	149
9	Large	Female	10	Axial	Rib	Chondroblastic	No	2	Yes	Yes	4	240	OS	102	Low	77	Low	48
10	Large	Female	11	Appendicular	Radius	Osteoblastic	No	2	Yes	Yes	1	485	OS	183	High	183	High	35
11	Large	Female	8	Axial	Jaw	Osteoblastic	No	1	No	No	0	83	OS	270	High	165	High	N/A
12	Small	Male	10	Axial	Rib	Osteoblastic	Yes	2	Yes	Yes	5	71	Euthanized because of OS	251	High	125	Low	163
13	Small	Male	11	Axial	Rib	Fibroblastic	Yes	2	No	No	0	4	Post-surgical complication	141	Low	130	Low	N/A
14	Large	Female	10	Appendicular	Tibia	Osteoblastic	No	1	Yes	Yes	1	240	Euthanized because of OS	161	High	100	Low	N/A
15	Large	Male	8	Appendicular	Humerus	Chondroblastic	No	1	No	No	0	150	OS	168	High	100	Low	N/A
16	Large	Female	9	Axial	Jaw	Osteoblastic	No	1	No	No	0	157	Euthanized because of OS	161	High	143	Low	N/A
17	Large	Male	8	Appendicular	Humerus	Chondroblastic	No	1	No	No	0	65	OS	125	Low	107	Low	N/A

**18**	**Large**	**Male**	**14**	**Appendicular**	**Femur**	**Osteoblastic**	**No**	**2**	**Yes**	**No**	**0**	**115**	**Euthanized because of OS**	**156**	High	**171**	High	**168**
**19**	**Large**	**Male**	**9**	**Appendicular**	**Ilium**	**Chondroblastic**	**Yes**	**1**	**No**	**No**	**0**	**1**	**Post-surgical complication**	**256**	High	**100**	Low	**N/A**
**20**	**Large**	**Male**	**9**	**Appendicular**	**Radius**	**Chondroblastic**	**No**	**1**	**No**	**No**	**0**	**104**	**Euthanized because of OS**	**131**	Low	**119**	Low	**N/A**
**21**	**Large**	**Female**	**7**	**Appendicular**	**Radius**	**Osteoblastic**	**No**	**2**	**Yes**	**Yes**	**1**	**277**	**OS**	**109**	Low	**91**	Low	**N/A**
**22**	**Small**	**Male**	**8**	**Axial**	**Jaw**	**Osteoblastic**	**No**	**2**	**No**	**No**	**0**	**21**	**OS**	**191**	High	**117**	Low	**N/A**
**23**	**Large**	**Female**	**4**	**Appendicular**	**Humerus**	**Chondroblastic**	**No**	**2**	**Yes**	**Yes**	**5**	**96**	**Euthanized because of OS**	**175**	High	**122**	Low	**83**
**24**	**Large**	**Male**	**12**	**Appendicular**	**Tibia**	**Chondroblastic**	**No**	**1**	**No**	**No**	**0**	**150**	**OS**	**183**	High	**178**	High	**N/A**
**25**	**Large**	**Male**	**13**	**Axial**	**Jaw**	**Fibroblastic**	**No**	**1**	**Yes**	**Yes**	**5**	**180**	**Still alive**	**135**	Low	**175**	High	**125**
**26**	**Large**	**Male**	**6**	**Appendicular**	**Femur**	**Fibroblastic**	**No**	**2**	**Yes**	**Yes**	**5**	**470**	**OS**	**113**	Low	**92**	Low	**N/A**
**27**	**Small**	**Female**	**12**	**Appendicular**	**Humerus**	**Osteoblastic**	**No**	**1**	**Yes**	**Yes**	**5**	**155**	**OS**	**203**	High	**174**	High	**N/A**
**28**	**Large**	**Female**	**15**	**Axial**	**Jaw**	**Fibroblastic**	**No**	**1**	**Yes**	**Yes**	**5**	**43**	**OS**	**213**	High	**175**	High	**N/A**
**29**	**Small**	**Female**	**9**	**Appendicular**	**Scapula**	**Osteoblastic**	**No**	**2**	**No**	**No**	**0**	**27**	**Euthanized because of OS**	**238**	High	**164**	High	**N/A**
**30**	**Large**	**Male**	**11**	**Appendicular**	**Humerus**	**Fibroblastic**	**No**	**1**	**Yes**	**Yes**	**5**	**578**	**OS**	**167**	High	**158**	High	**N/A**
**31**	**Small**	**Male**	**10**	**Appendicular**	**Humerus**	**Osteoblastic**	**No**	**2**	**Yes**	**Yes**	**5**	**26**	**OS**	**240**	High	**160**	High	**N/A**
**32**	**Large**	**Male**	**13**	**Axial**	**Jaw**	**Osteoblastic**	**No**	**2**	**No**	**No**	**0**	**21**	**Euthanized because of OS**	**228**	High	**187**	High	**N/A**
**33**	**Large**	**Male**	**6**	**Appendicular**	**Femur**	**Osteoblastic**	**No**	**2**	**No**	**No**	**0**	**17**	**Euthanized because of OS**	**204**	High	**136**	Low	**N/A**
**34**	**Large**	**Male**	**3**	**Axial**	**Rib**	**Chondroblastic**	**No**	**2**	**No**	**No**	**0**	**11**	**Euthanized because of OS**	**213**	High	**172**	High	**N/A**
**35**	**Large**	**Female**	**9**	**Appendicular**	**Radius**	**Osteoblastic**	**No**	**1**	**No**	**No**	**0**	**17**	**Euthanized because of OS**	**210**	High	**139**	Low	**N/A**
**36**	**Large**	**Female**	**9**	**Appendicular**	**Humerus**	**Osteoblastic**	**No**	**2**	**Yes**	**Yes**	**5**	**270**	**OS**	**165**	High	**140**	Low	**N/A**
**37**	**Large**	**Female**	**13**	**Appendicular**	**Scapula**	**Chondroblastic**	**No**	**2**	**No**	**No**	**0**	**135**	**OS**	**143**	Low	**102**	Low	**N/A**
**38**	**Large**	**Male**	**12**	**Axial**	**Vertebrae**	**Osteoblastic**	**No**	**1**	**Yes**	**No**	**0**	**54**	**OS**	**218**	High	**112**	Low	**N/A**
**39**	**Large**	**Female**	**9**	**Appendicular**	**Ilium**	**Osteoblastic**	**No**	**2**	**No**	**No**	**0**	**3**	**Post-surgical complication**	**194**	High	**150**	High	**N/A**
**40**	**Small**	**Male**	**13**	**Axial**	**Jaw**	**Osteoblastic**	**No**	**2**	**No**	**No**	**0**	**47**	**Euthanized because of OS**	**298**	High	**270**	High	**N/A**
**41**	**Large**	**Female**	**9**	**Appendicular**	**Humerus**	**Chondroblastic**	**No**	**2**	**Yes**	**Yes**	**5**	**76**	**OS**	**245**	High	**179**	High	**107**
**42**	**Large**	**Male**	**6**	**Appendicular**	**Radius**	**Osteoblastic**	**No**	**1**	**Yes**	**Yes**	**1**	**82**	**Euthanized because of**	**278**	High	**148**	Low	**N/A**

													OS					
43	Large	Female	11	Appendicular	Tibia	Osteoblastic	No	2	Yes	Yes	1	288	Euthanized because of OS	169	High	273	High	N/A
44	Large	Female	11	Appendicular	Humerus	Osteoblastic	No	1	Yes	Yes	5	90	Euthanized because of OS	240	High	255	High	N/A
45	Large	Female	7	Axial	Skull	Osteoblastic	No	1	No	No	0	24	Euthanized because of OS	254	High	214	High	N/A
46	Large	Male	8	Appendicular	Humerus	Osteoblastic	Yes	2	No	No	0	14	Euthanized because of OS	262	High	222	High	100
47	Small	Male	14	Axial	Jaw	Osteoblastic	No	2	No	No	0	154	Still alive	248	High	228	High	N/A
48	Large	Male	7	Appendicular	Femur	Osteoblastic	No	1	No	No	0	7	Post-surgical complication	278	High	283	High	N/A
49	Large	Male	11	Appendicular	Femur	Fibroblastic	No	1	Yes	Yes	5	655	OS	145	Low	164	High	50
50	Large	Male	7	Appendicular	Humerus	Osteoblastic	No	1	No	No	0	142	Still alive	229	High	220	High	56

Supplementary Table S1. Clinicopathological and immunohistochemical data for the 50 dogs with OS included in the survival analysis (ASAP-50).

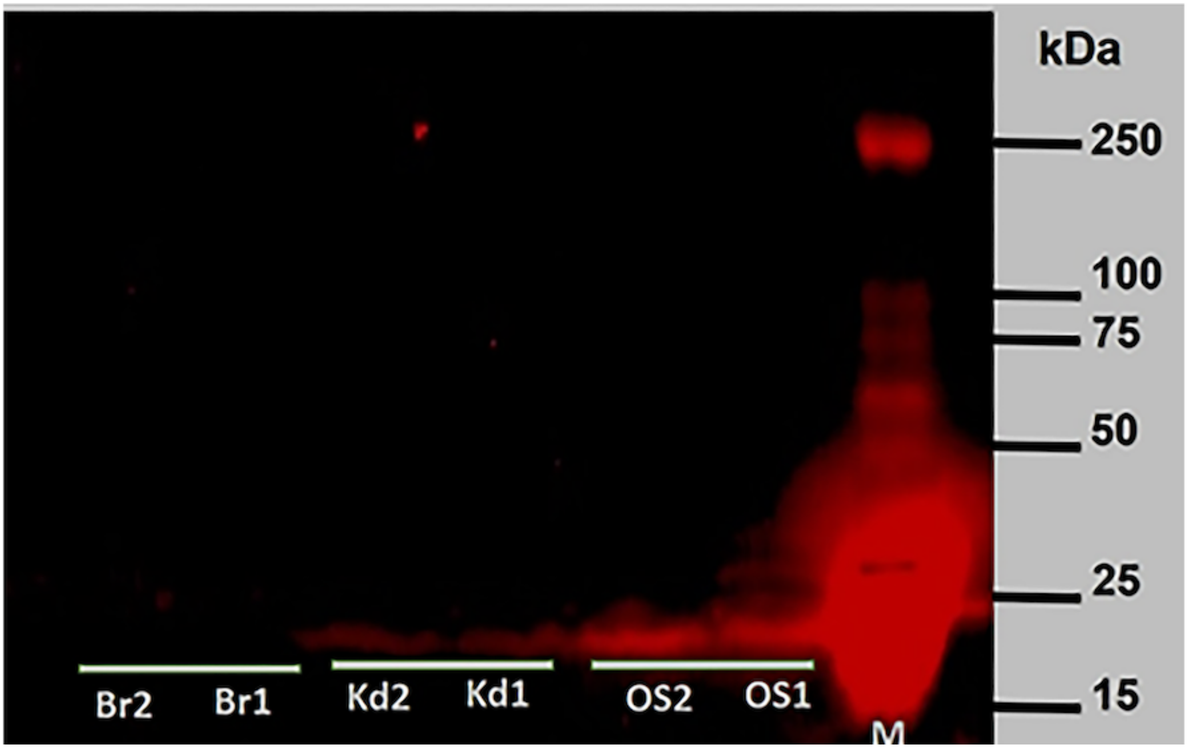

Supplementary Figure S1. Detection of PTHrP protein in canine OS tissues using western blot. OS1 & OS2 are two different cases of canine chondroblastic OS. Kd1 & Kd2 are two samples of normal canine kidney tissue (positive control) and Br1 & Br2 are brain cortex samples from two normal beagles. Expected molecular weight for PTHrP is 20 kDa.

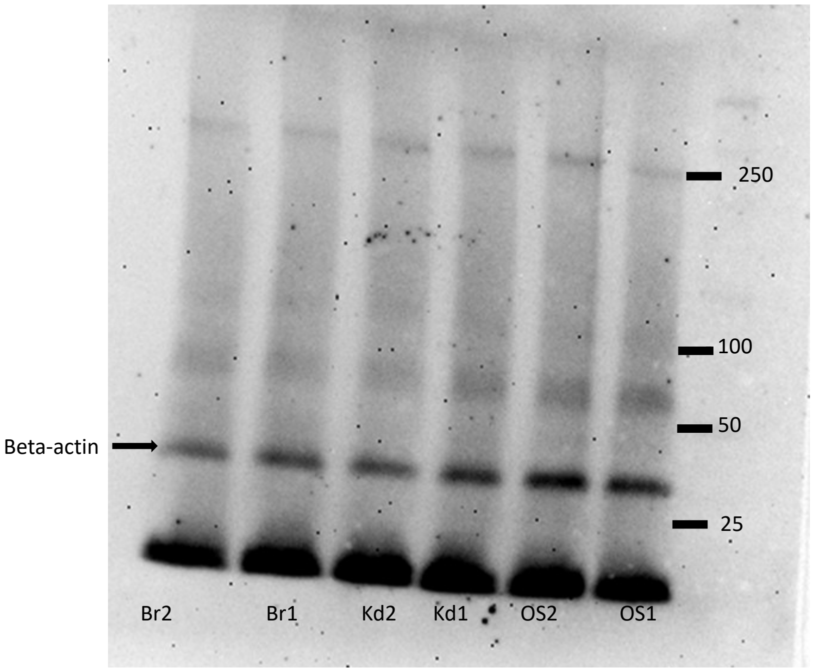

Supplementary Figure S2. The protein loading control (beta-actin) for the western blot in the upper panel. The beta-actin antiserum is a rabbit monoclonal (SP124, Thermofisher Scientific, Scoresby, Australia) at 1:200 dilution. The expected molecular weight for beta-actin is
